# Percutaneous Coronary Intervention in an Extremely Rare Case of Double Circumflex Coronary Arteries With Acute Myocardial Infarction

**DOI:** 10.7759/cureus.23073

**Published:** 2022-03-11

**Authors:** Navdeep S Sidhu, Sumandeep Kaur

**Affiliations:** 1 Cardiology, Guru Gobind Singh Medical College and Baba Farid University of Health Sciences, Faridkot, IND; 2 Nursing Sciences, Baba Farid University of Health Sciences, Faridkot, IND

**Keywords:** congenital coronary anomalies, congenital anomalies of coronary arteries, primary percutaneous coronary intervention (pci), right circumflex artery, twin circumflex artery, double circumflex artery, "anomalous coronary artery", anomalous left circumflex artery, acute myocardial infraction

## Abstract

The anomalous origin of the left circumflex (Cx) artery (LCX) from the right coronary sinus or the right coronary artery (RCA) has been reported as one of the most common congenital coronary anomalies. However, the occurrence of double or twin Cx coronary arteries has been sparsely reported in the literature. We describe a rare case of a middle-aged male with acute myocardial infarction (MI) who had double Cx coronary arteries, one arising from the RCA and the other from the left main coronary artery. He underwent successful angioplasty with the stenting of the culprit right Cx artery (RCX).

## Introduction

Congenital coronary anomalies are rare clinical entities with a reported incidence of 0.6-5.4% [1,2]. Most of these are detected as incidental findings during coronary angiography (CAG). An anomalous circumflex (Cx) coronary artery arising from the right coronary sinus or the right coronary artery (RCA) is one of the most frequent coronary anomalies, seen in 34.4-57.9% of the patients with coronary anomalies [3,4]. Double Cx coronary arteries, with one originating from the left system and the other from the right system, is an exceptionally rare coronary anomaly that has been reported only a few times in the literature worldwide [5-20]. We present a case of double Cx arteries in a middle-aged male with acute myocardial infarction (MI), and its successful management using percutaneous coronary intervention (PCI) of the diseased right Cx artery (RCX).

## Case presentation

**Figure 1:**
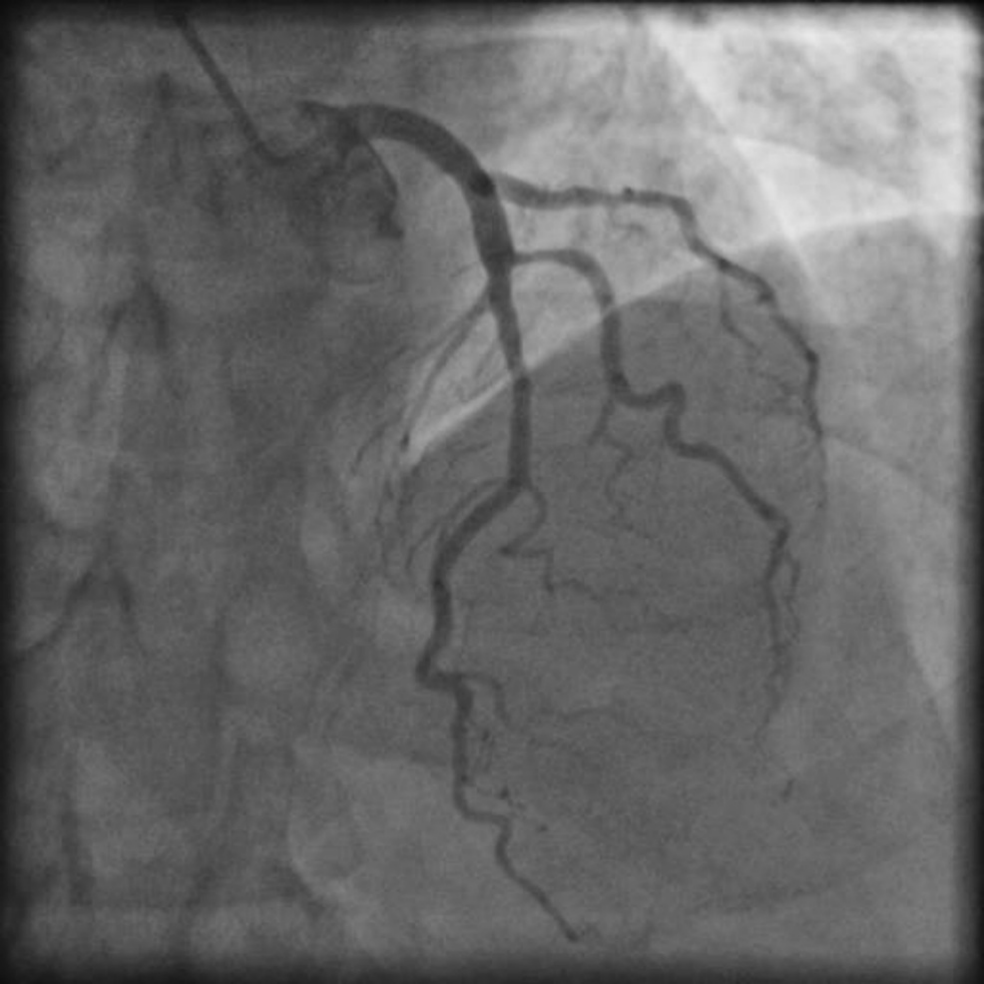
Coronary angiogram in posterior-anterior (PA) cranial projection showing plaquing in mid-left anterior descending (LAD) and intermediate stenosis in the major diagonal branch

A 50-year-old male presented to our institution with a history of diffuse retrosternal chest pain of three hours' duration. The patient was a known hypertensive for the last two years, but on irregular treatment. He was a non-smoker, non-diabetic, and had no history of dyslipidemia or any family history of premature coronary artery disease (CAD). On presentation, he had a pulse rate of 88 beats per minute (bpm), blood pressure of 140/90 mmHg, and unremarkable auscultation of the cardiovascular and respiratory systems. The ECG showed non-specific ST-T changes, and his hemogram and renal function tests were within the normal range. He had markedly elevated levels of troponin I at 0.9 ng/ml (normal level: <0.1 ng/ml). The 2-D echocardiogram showed hypokinesia of basal and mid-inferolateral segments with an ejection fraction of around 50%. The patient was administered loading doses of aspirin, ticagrelor, and atorvastatin and was shifted to the catheterization laboratory for an early invasive procedure in light of the ongoing anginal symptoms and significantly elevated troponin levels. CAG performed using the right radial arterial approach revealed a normal left main coronary artery dividing into the left anterior (LAD) and left Cx arteries (LCX). The LAD had an insignificant disease in the mid-segment and there was intermediate stenosis in a large diagonal branch (Figure 1).

The LCX had an early exit from the left atrioventricular groove and continued as the first obtuse marginal (OM) branch, which had insignificant plaquing in its proximal part (Videos 1, 2).

**Video 1:** Coronary angiogram in posterior-anterior (PA) caudal view showing early exit of left circumflex artery (LCX) from the left atrioventricular groove and its continuation as first obtuse marginal (OM) artery

**Video 2:** Coronary angiogram in right anterior oblique (RAO) caudal view showing early exit of left circumflex artery (LCX) from the left atrioventricular groove and its continuation as first obtuse marginal (OM) artery

**Figure 2:**
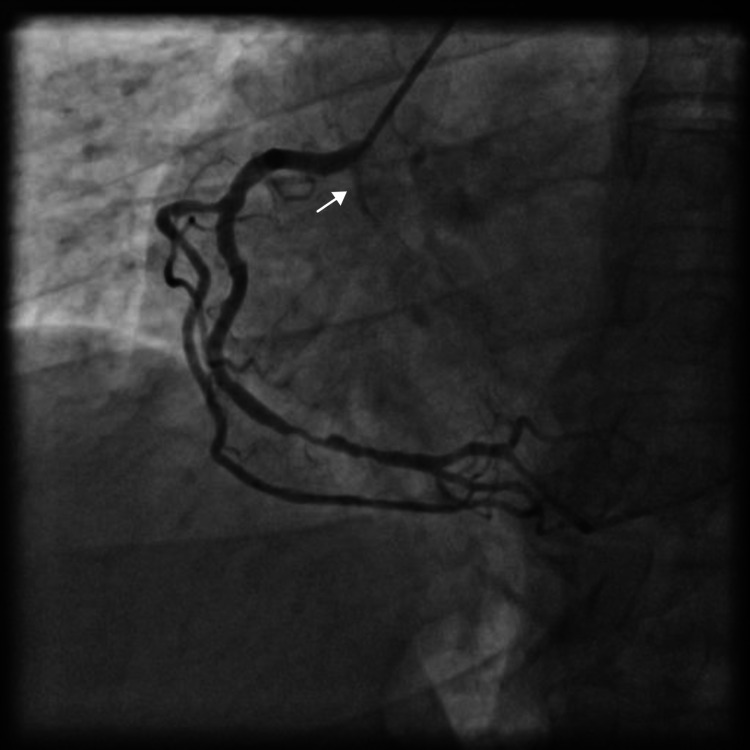
Left anterior oblique (LAO) view coronary angiogram of the right coronary artery (RCA) demonstrating severe disease in distal RCA and faintly seen occluded anomalous right circumflex artery (RCX) (arrow)

**Figure 3:**
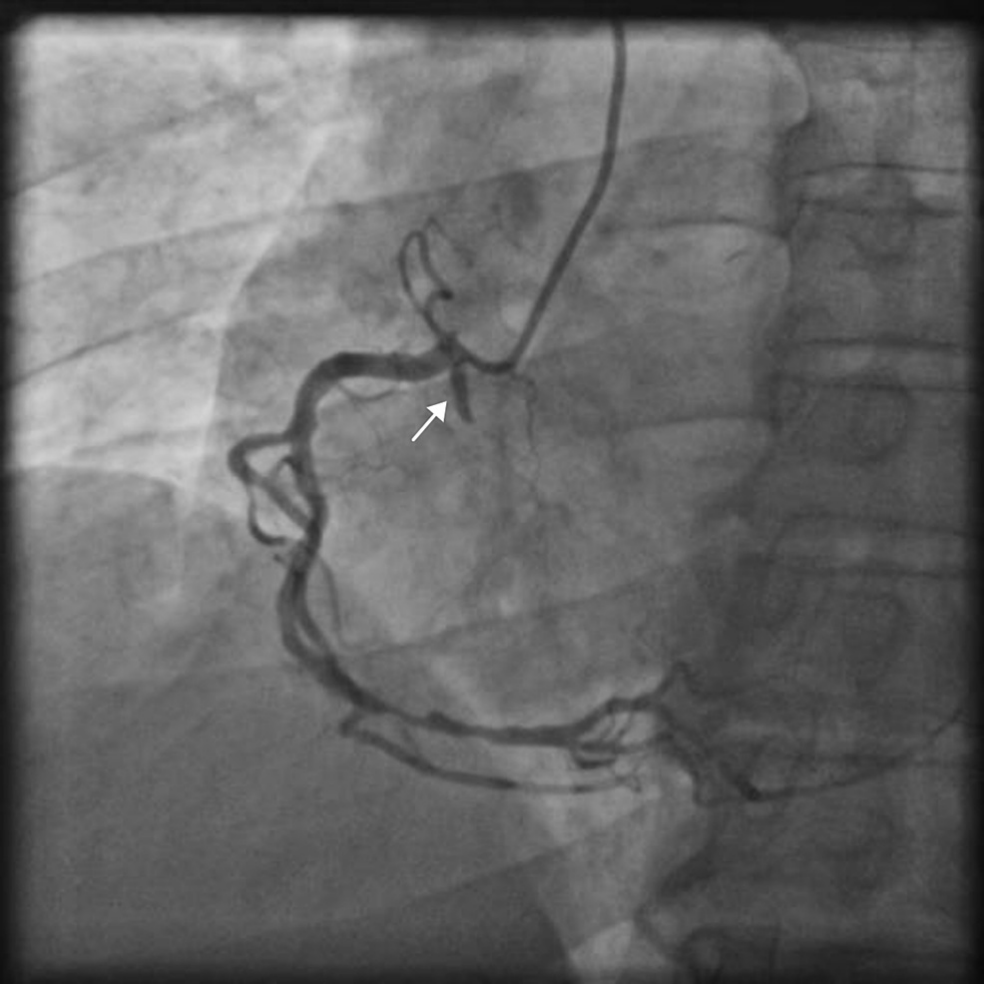
Coronary angiogram of the right coronary artery (RCA) in left anterior oblique (LAO) projection after withdrawal of the catheter, clearly demonstrating the anomalous right circumflex artery (RCX) with proximal thrombotic occlusion (arrow)

The angiogram of the right system revealed a dominant RCA with mid-segment plaquing and a distal diffuse 85-90% stenosis with thrombolysis in MI (TIMI) III flow. On closer look, an RCX was seen arising from the ostio-proximal part of the RCA (Figure 2, Video 3), which was more evident on slight withdrawal of the catheter from the RCA (Figure 3, Video 4).

**Video 3:** Left anterior oblique (LAO) view coronary angiogram of the right coronary artery (RCA) demonstrating severe disease in distal RCA and faintly seen occluded anomalous right circumflex artery (RCX)

**Video 4:** Coronary angiogram of the right coronary artery (RCA) in left anterior oblique (LAO) projection after withdrawal of the catheter, clearly demonstrating the anomalous right circumflex artery (RCX) with proximal thrombotic occlusion

**Figure 4:**
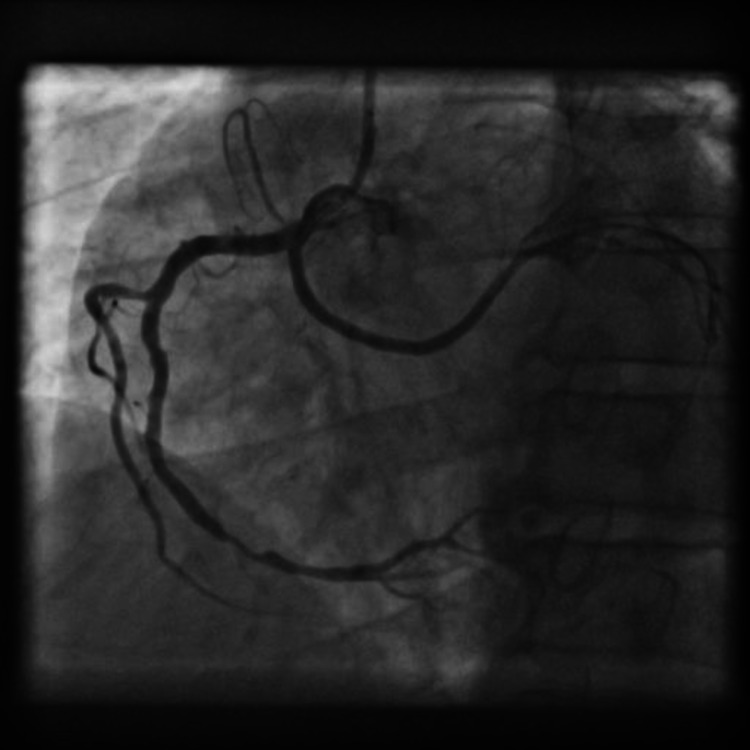
Coronary angiogram of the right system in left anterior oblique (LAO) projection after successful angioplasty of the anomalous right circumflex artery (RCX)

This RCX had proximal thrombotic occlusion and was the probable cause of the patient’s symptoms. We immediately proceeded with the PCI of the occluded RCX. An AR1 guide catheter was used to engage the anomalous RCX after minimal manipulation, and a BMW guidewire (Abbott Laboratories, Chicago, IL) was used to cross the lesion. After performing the thrombosuction, we pre-dilated the lesion with a 2.25 x 10-mm semi-complaint balloon. Two overlapping drug-eluting stents (DES) were implanted (2.5 x 28 mm and 2.5 x 18 mm) and post-dilatation was performed with a non-compliant 2.5 x 15-mm balloon, resulting in TIMI III flow without any local complications (Figure 4).

During the same procedure, PCI to the distal RCA was performed with 3.5 x 33-mm DES, using the same guide catheter and guidewire (Video 5).

**Video 5:** Left anterior oblique (LAO) caudal view coronary angiogram of the right coronary artery (RCA) after successful angioplasty of the RCA

The patient had an eventful recovery after the procedure and was discharged after two days in a stable condition. Currently, the patient is asymptomatic after a follow-up period of one year.

## Discussion

Coronary anomalies constitute a relatively rare, diverse group of congenital anomalies of coronary artery origin, course, or termination with varied clinical presentations. Although these anomalies are most often detected incidentally during angiography, some of these anomalies can be associated with serious clinical presentations including MI, arrhythmias, syncope, or sudden cardiac death [1,2]. While performing CAG, a high index of suspicion should be maintained about the possible existence of a coronary anomaly, especially when the symptoms of a patient are not easily explained by the disease in the normally located coronary arteries, as illustrated by our case. Moreover, the operator should be aware of the need for the manipulation of the catheter to clearly define the origin and course of an anomalous coronary artery, to avoid errors in the management of a patient. An anomalous Cx artery arising from the right sinus or from the ostio-proximal part of the RCA can easily be missed as the 5F diagnostic catheters used in contemporary clinical practice for CAG through the radial route are easily engaged deeply into the proximal part of the large-diameter RCA seen in most patients.

Although the anomalous origin of LCX has been described to be one of the most common congenital anomalies in many large angiographic series [3-4], the occurrence of double or twin Cx coronary arteries has been reported very rarely in the literature [5-20]. In most of the reported cases of double Cx arteries, one Cx artery (LCX) had its origin from the left main artery while the other (RCX) had its origin from the right coronary sinus or the RCA [5-18]. A similar finding was seen in our case with the LCX arising from the left main and the RCX arising from the ostio-proximal part of the RCA. In extremely rare cases, the two Cx arteries may have their origin from the left coronary sinus, as reported by Warner et al. and Tekbas et al. [19,20].

In our case, the anomalous RCX with proximal thrombotic occlusion was probably responsible for the acute MI and the ongoing chest pain, as the RCA (although a diseased vessel) with TIMI III flow at rest could not account for the ongoing anginal symptoms at rest. Hence, we proceeded with the PCI of this anomalous vessel, with a plan to perform PCI of the diseased RCA as well in the same sitting depending upon the patient’s condition and the amount of contrast volume used. An AR guide catheter provided sufficient support for the PCI of the RCX after slight manipulation and the PCI could be completed with the use of routine hardware and about 90 ml of the contrast. The PCI of the diseased RCA was also performed in the same sitting with the use of the same hardware and an additional 70 ml of contrast volume. Although adequate support was provided by an AR guide catheter in our case, some other potentially useful maneuvers to enhance the guide support during the PCI of such tortuous anomalous Cx arteries include the use of AL or Voda catheter, the use of buddy wire technique, or the use of guide catheter extensions.

Our case is an exceptionally rare case of a PCI of anomalous RCX in the presence of double Cx arteries; to the best of our knowledge, only three such cases have been reported previously in the literature worldwide [14,16,18]. Otlu et al. reported a case of twin Cx arteries originating from the left and right coronary systems in a patient with recent-onset angina and successfully performed PCI to the diseased RCX artery using the trans-radial approach [14]. In a case reported by Sinha et al., successful primary PCI was performed to the culprit anomalous RCX in a patient with acute inferior wall MI [16]. Uğuz et al. have reported a case of a middle-aged male with acute MI where twin Cx arteries were noted on CAG and successful PCI to the RCX was performed [18].

The most important clinical problem in the case of double Cx arteries is the chance of missing an anomalous RCX, especially if close attention is not paid to the clinical status of the patient and careful scrutiny of the angiograms is not done, as the deep engagement of the catheter in the RCA may often impair proper visualization of the anomalous RCX arising from the right sinus or the ostial part of RCA. A significant limitation of our case report is that we could not get a CT CAG to better define the anomalous origin and course of the RCX due to the financial constraints of the patient.

## Conclusions

Treating physicians should be aware of the various types of congenital coronary anomalies as this knowledge is very essential to avoid missing a culprit coronary artery or causing an inadvertent injury during coronary intervention or cardiac surgery. The occurrence of double or twin Cx coronary arteries in an extremely rare congenital coronary anomaly. PCI can be safely performed in cases of diseased anomalous Cx coronary arteries, although maneuvers to gain additional guide support may sometimes be required.
